# AdoR-1 (Adenosine Receptor) Contributes to Protection against Paraquat-Induced Oxidative Stress in *Caenorhabditis elegans*

**DOI:** 10.1155/2022/1759009

**Published:** 2022-12-22

**Authors:** Chunyan Ling, Liangcheng Shang, Xin Xie, Sudan Ye, Ningjing Wang, Chun Chen

**Affiliations:** ^1^China Jiliang University, College of Life Sciences, Engineering Training Centre/College of Innovation, Hangzhou 310018, China; ^2^Zhejiang Institute of Economics and Trade, Hangzhou 310018, China

## Abstract

AdoR-1, the single adenosine receptor homolog in *Caenorhabditis elegans*, which belongs to the superfamily of G-protein coupled receptors (GPCRs), mediates most of the physiological effects of extracellular adenosine. Adenosine has been proved to improve the survival rate of *C. elegans* in oxidative stress conditions. However, the potential mechanism of adenosine's protective effect against oxidative stress via AdoR-1 has not been studied. In this study, *C. elegans* were divided into three groups: two groups with paraquat treatment, one in the presence and one in the absence of adenosine, and an untreated control group. Results indicate that many differentially expressed genes were found to be enriched significantly in neural-related signaling pathways among transcriptome data of three groups. Further gene network analysis showed that some important genes well known to be involved in promoting the acetylcholine release pathway, such as *dop-1*, *egl-30*, and *unc-13,* and those involved in promoting the neuropeptide release pathway, such as *kin-1,* were upregulated by paraquat induction but downregulated after adenosine treatment. Meanwhile, a completely opposite trend was observed for the *goa-1* gene that inhibits the acetylcholine-release and neuropeptide-release pathway. Additionally, some biochemical assays including SOD, GSSG, GSH, and AChE were measured to identify the potential protection of adenosine against oxidative stress between wild-type strain N2 and *ador-1* gene knockout strain EG6890. Conclusively, our study revealed series of adenosine receptor-mediated genes in *C. elegans* that might act as regulators of paraquat-induced oxidative stress and may indicate adenosine's promising protective effects.

## 1. Introduction

It is well documented that adenosine (ADO) has an impact on the treatment of pathological conditions, such as oxidant injury in neural cells, that functions through adenosine receptor-mediated signaling pathways [1]. AdoRs, which belong to the superfamily of G-protein coupled receptors (GPCRs), mediate most of the physiological effects of extracellular adenosine in vertebrates and invertebrates [2]. Currently, four distinct AdoR isoforms have been identified in mammals. Two of these have been positively coupled to adenylate cyclase (A2A and A2B), and the other two (A1 and A3) negatively regulate this enzyme [3].

The first completely sequenced genome of a multicellular organism was that of *Caenorhabditis elegans*, and many genes and signaling pathways have been shown to be conserved between nematodes and humans [4]. In particular, G-protein pathways such as the dopamine, serotonin, 5-HT, and other neurotransmitter signaling pathways, as well as nerve signaling pathways, have been shown to be highly similar between *C. elegans* and vertebrates [5]. AdoR-1, the single adenosine receptor homolog in *C. elegans* that is expressed by the *ador-1* gene, is known to be an ortholog of human *A2BR* (adenosine A2b receptor).

Previous evidence has shown that Drosophila AdoR mutants have significantly increased sensitivity to the oxidative stress caused by paraquat [6]. Our lab's latest studies also found that a certain concentration of adenosine may improve the survival rate of *C. elegans* in oxidative stress conditions [7] However, AdoR-1 has not been characterized in terms of expression or function, and adenosine might generally be classified as a neurotransmitter in any system and has been shown to have a protective effect against oxidative stress [8]. Thus, it seems that AdoR-1 might be a potential target that may predictably activate the neuropeptide-related receptor activity involved in neuropeptide signaling pathways.

Paraquat (PQ), also known as 1,1′-dimethyl-4,4′-bipyridinium dichloride, is a common chemical substance used for generating an oxidative stress in the laboratory, particularly when used to treat the nematode *C. elegans* [9, 10]. Therefore, in the present study, transcriptome data from *C. elegans* groups treated with paraquat in the presence or absence of adenosine were analyzed to explore gene expression during paraquat-induced oxidative stress, and the biochemical assays from wild-type and *ador-1* gene knockout strains treated with paraquat in the presence or absence of adenosine were also measured to evaluate the protective effect of AdoR-1. The results were used to uncover the molecular mechanism driving the protective effect of the nematode AdoR-1 after the addition of adenosine in these conditions.

## 2. Materials and Methods

### 2.1. Strain and Chemicals Solution Preparation

The *C. elegans* used in the present study were wild-type Bristol (N2), originally obtained from the Caenorhabditis Genetic Center (Minneapolis, USA). They were maintained on nematode growth medium (NGM) plates seeded with *Escherichia coli* OP50 at 20°C as described in a previous study [11].

Paraquat and adenosine (supplied by Sigma-Aldrich; Merck KGaA, Germany) were dissolved in 10 mL sterilized M9 buffer (Na_2_HPO_4_, 6 g, KH_2_PO_4_, 3 g, NaCl, 5 g, and MgSO_4_·7H_2_O, 0.25 g) based on a previous study, where the final treatment concentrations were 150 mM and 3,000 *μ*M, respectively [7].

### 2.2. *C. elegans* Treatment and Grouping

A mixed-population plate with gravid adults that had laid plenty of eggs was used to directly harvest eggs. Eggs were then transferred to fresh plates using a sterile pick for incubation at 20°C. Age-synchronous populations of L4-larval nematodes were obtained at 20°C for ~48 h as described in a previous study [12]. Approximately 1,000 age-synchronized young adults were treated individually with paraquat (150 mM) and paraquat in the presence of adenosine (3,000 *μ*M), which were abbreviated as the PQ and PQ_AD groups, respectively. Sterilized M9 buffer solution treatment was used as a control (CK group). Three groups of nematodes were then treated with the corresponding conditions for 5 minutes prior to washing with M9 buffer solution three times to remove the bulk of residual bacteria. Then, the samples were transferred to 1.5 mL tubes, and excess supernatant was removed via centrifugation (2,000 × g, 5 min). The samples were frozen immediately in liquid nitrogen for 5 min and then stored at −80°C. Total RNA was extracted immediately using TRIzol (Life Technologies Corporation, Carlsbad, USA) according to the manufacturer's protocol.

### 2.3. RNA Transcriptome Sequencing Analysis

RNA transcriptome sequencing analysis was performed by BGI Genomics Corporation (Beijing, China) using Illumina HiSeq technology. Reference genome and gene model annotation files were downloaded from WormBase (WS269). To assess molecular signaling pathway changes after paraquat treatment in the presence or absence of adenosine in *C. elegans*, the data were divided into two comparisons: CK versus PQ (comparison A) and PQ versus PQ_AD (comparison B). An index of the reference genome was then constructed using Hisat2 v2.0.5, and paired-end clean reads were aligned to this reference genome using Hisat2 v2.0.5 [13–15]. FeatureCounts v1.5.0-p3 was used to count the read numbers mapped to each gene [16]. Then, the FPKM of each gene was calculated based on the length of each gene and the read count mapped to these genes. Differential expression analysis of these two groups was then performed using the R package DESeq2 (1.16.1) based on the negative binomial distribution [17]. The Benjamini–Hochberg approach was used to modulate the resulting *P* values to control the false discovery rate. Corrected values of FGR ≤ 0.001 and fold change ≥ 2 were set as the threshold for significantly differential expression. GO enrichment analysis of differentially expressed genes was achieved using the clusterProfiler R package with an adjusted value of *P* < 0.05 [18].

### 2.4. Quantitative Reverse-Transcription PCR (qRT-PCR)

Based on a comparison of key differential genes in CK vs. PQ and PQ vs. PQ_AD from our transcriptome data, qRT-PCR was used to verify the expression of differential genes from our GO enrichment analysis and the mRNA expression of G-protein signaling pathway genes. Primers for each gene and the PCR procedure parameters are listed in Tables 1 and 2, respectively. Primer design was performed with available sequences using the Primer-BLAST software (https://www.ncbi.nlm.nih.gov/tools/primer-blast/), and primer synthesis was performed by Sangon Bioengineering Co., Ltd. (Shanghai, China). Total RNA was used to synthesize cDNA with the PrimeScript™ RT reagent Kit with gDNA Eraser (Takara Bio, Japan). Single-stranded cDNA was amplified by comparative qRT-PCR using Takara TB GREEN Premix Ex Taq II reagent (Takara Bio, Japan) on a Bio-Rad CFX 384 real-time PCR instrument. Eleven differential genes in our GO enrichment and six genes from the G-protein signaling pathway were analyzed using CFX maestro Version 4.1 (Bio-Rad, Hercules, CA, USA). Gene expression levels were calculated using the 2^−ΔΔCt^ method [19]. The ΔCt value of each sample was calculated using *act-1* as an endogenous control gene. Student's *t*-test was performed to statistically compare the gene expression of qRT-PCR results.

### 2.5. GO Enrichment Analysis

Differential gene lists in the comparison of CK vs. PQ vs. PQ_AD were examined using Gene Ontology term enrichment by GOrilla (http://cbl-gorilla.cs.technion.ac.il/) using default parameters.

### 2.6. Protein-Protein Interaction (PPI) Network Comparison Using the STRING Database

Interaction evidence of the AdoR-1 protein from the STRING database v11.5 [20] was used (scores ranging from 0 to 1000, retrieved from https://string-db.org on 12/19/2021). STRING transmits experimental and predictive interaction-founded information, and the interaction generated via the online tool is defined with 3-dimensional structures, auxiliary information, and confidence scores. The confidence score in the current study was set as 0.900, utilizing the STRING platform.

### 2.7. Measurement of SOD, GSH, GSSG, and AChE between Wild-Type Strain N2 and Ador-1 Gene-Knockout Strain EG6890

In order to prove the effect of *ador-1* gene during the PQ and PQ_AD treatment on the *C. elegans*, EG6890 strain, *ador-1*(ox489), kindly supplied from Dr. Erik Jorgensen laboratory (University of Utah, USA), was evaluated for its biochemical activity with wild-type N2. The EG6890 strain has a deletion from 1 kb upstream and the first three exons of the *ador-1* gene and was outcrossed six times. The synchronized nematodes treated individually with same above conditions (150 mM paraquat and paraquat in the presence of 3,000 *μ*M adenosine) were harvested and washed free from bacteria using M9 buffer as described above.

An extract of nematodes was prepared by ground in liquid nitrogen with sterile mortar and pestle, repeated freezing and thawing twice. Homogenates were centrifuged at 12,000 × g, 4°C for 10 min, and the supernatant fractions were assayed for protein concentration (BCA kit, Beyotime Biotechnology, China). Superoxide dismutase (SOD), reduced glutathione (GSH), oxidized glutathione (GSSG), and acetylcholinesterase (AChE) activities were detected using commercial kits (Nanjing Jiancheng Bioengineering Institute, Nanjing, China) according to the manufacturer's instruction. All experiments were performed in triplicate.

## 3. Results

### 3.1. RNA Sequencing

A summary of data quality assessment results from our RNA-seq data is shown in Supplementary Table S1. After the sequencing data from the 3 groups of nematode samples were processed through the steps of assembly, quality assessment, screening, and filtering, the clean reads obtained from each group were all more than 40 M. The number of bases with a sequencing error rate of ≤0.1% (Q30) accounted for more than 85% of the total number of bases on average. These results suggested that the sequences and alignments were of good quality. This sequencing data were submitted to the BioProject database as PRJNA809866, and the website was http://www.ncbi.nlm.nih.gov/bioproject/809866.

### 3.2. Differential Gene Expression in RNA-Seq Data

**Figure 1:**
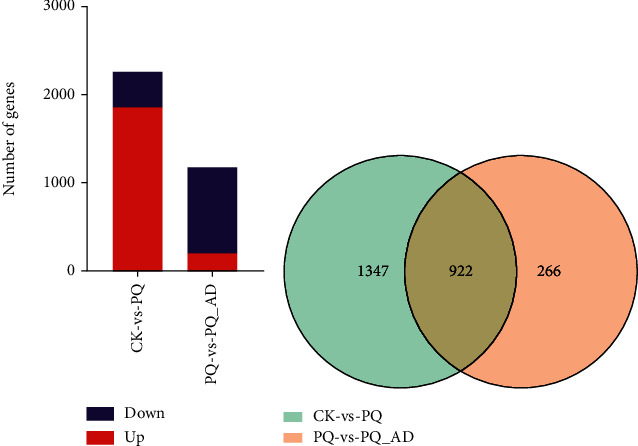
Statistics of differential gene expression in *C. elegans* RNA-seq data between CK vs. PQ and PQ vs. PQ_AD. (a) Number of differential genes including upregulation (red color) and downregulation (blue color). (b) Venn diagram of differentially expressed genes.

Using DESeq-based statistical inference, a total of 2,537 genes were found to be differentially expressed in pair-wise comparisons of two groups, including 1878 upregulated and 391 downregulated genes in a comparison of the CK group with the PQ group, and 213 upregulated and 975 downregulated genes in a comparison of the PQ group with the PQ_AD group, respectively (Figure 1(a)). Of these differentially expressed genes, 922 were found to be coregulated between CK vs. PQ and PQ vs. PQ_AD (Figure 1(b).

### 3.3. GO-Based Enrichment Analysis

**Figure 2:**
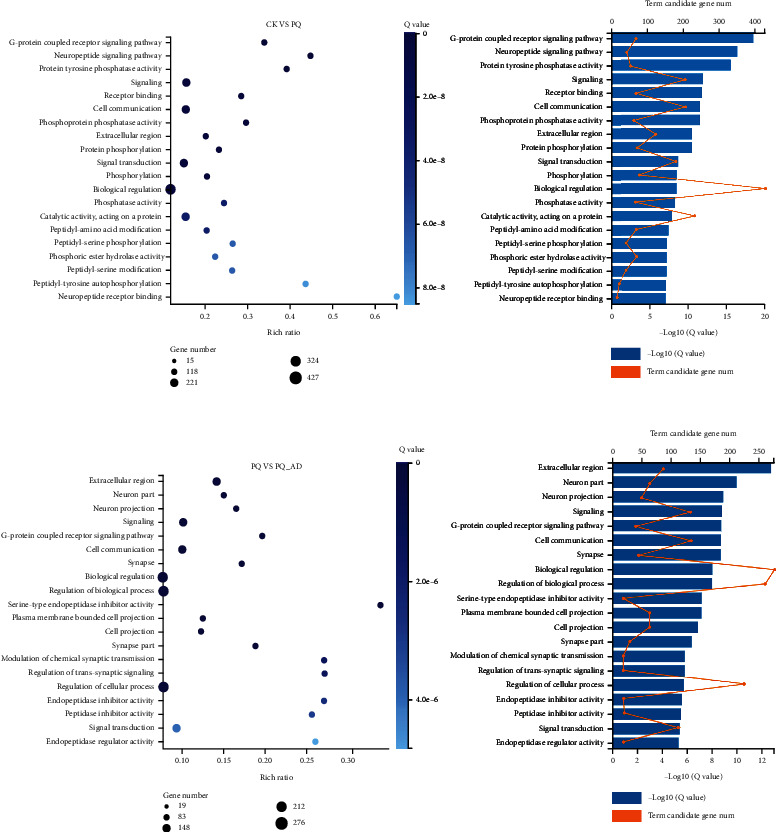
Top 20 GO term from GO enrichment of CK vs. PQ (a) and PQ vs. PQ_AD (b).

We next performed GO (gene ontology) analysis on the differentially expressed genes identified by RNA sequencing in the CK vs. PQ and PQ vs. PQ_AD groups (Figures 2(a) and 2(b)). We found that the GO term present in both groups was enriched in the following five categories: GPCR signaling pathway, signaling, extracellular region, cell communication, and cell signal transduction. In our enrichment results, the gene classes containing more than 3 genes in each GO term were defined as high-frequency gene classes. The obtained high-frequency gene families and their functions in the five GO classifications are shown in Supplementary Table S2 (All data based on WormBase). In this table, the differential genes were mainly clustered in neural-related function, including the *dop* gene family, the neuropeptide-related gene family (such as *flp*, *ins*, and *nlp*), the neuropeptide receptor gene family (such as *npr*), and the acetylcholine release-related *unc* gene family.

### 3.4. Validation of RNA-Seq Differential Genes by qRT-PCR

**Figure 3:**
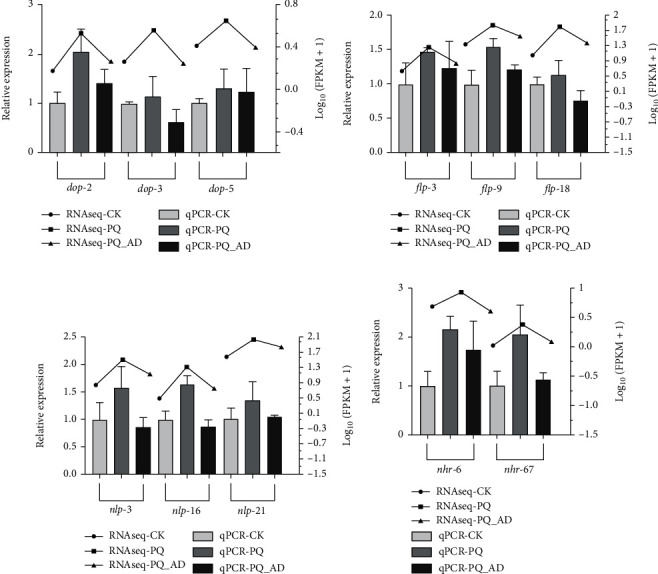
qRT-PCR verification of RNA-seq differential genes within four gene classes. Columns represent the relative expression of various groups based on qPCR data. Dotted lines represent the relative expression of various groups based on RNA-seq data. (a) *DOP* gene class, including *dop-2*, *dop-3,* and *dop-5*. (b) *FLP* gene class, including *flp-3*, *flp-9,* and *flp-18*. (c) *NLP* gene class, including *nlp-3*, *nlp-16,* and *nlp-21*. (d) *NHR* gene class, including *nhr-6* and *nhr-67*. Three replicates were used in qRT-PCR per treatment.

Eleven differential genes (listed in Table 1) were randomly selected from the above-mentioned high-frequency gene classes for qRT-PCR validation and were divided into four gene classes: *DOP* (DOPamine receptor), *FLP* (FMRF-like peptide), *NLP* (neuropeptide-like protein), and *NHR* (nuclear hormone receptor). From the overall trend, the qPCR verification results of the relative expression of differential genes were consistent with our RNA-seq results (Figure 3). The trends in the expression data from RNAseq and qRT-PCR were that genes were upregulated after PQ treatment and were reduced after the addition of adenosine, indicating that our RNA-seq results were reliable and accurate and could be used for subsequent analysis.

### 3.5. Validation of G-Protein Signaling Pathway Genes by qRT-PCR

**Figure 4:**
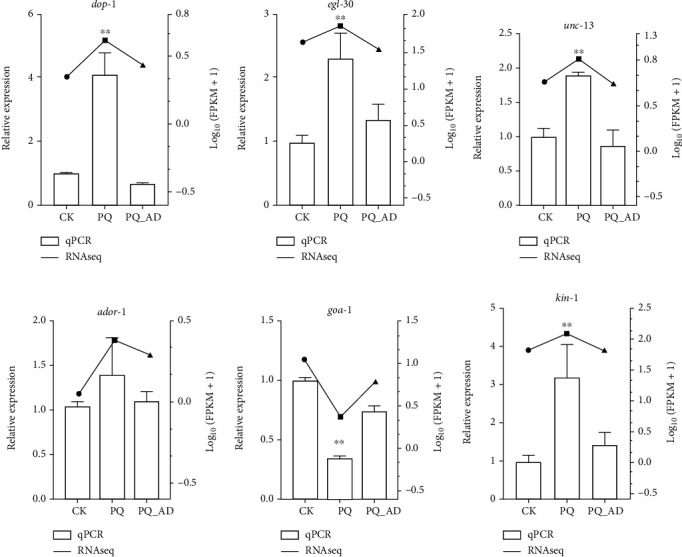
Transcriptome analysis of G protein signaling pathway genes and qPCR verification. Columns represent the relative expression of various groups based on qPCR. Dotted lines represent the relative expression of various groups based on RNA-seq data. Figures 4(a)–4(f) represent the qPCR data and RNA-seq data of *dop-1*, *egl-30*, *unc-13*, *ador-1*, *goa-1*, and *kin-1*, respectively. Three replicates were used per treatment. Differences compared to control group were considered significant at ^∗∗^*P* < 0.01.

The involved genes that were known to play a role in *C. elegans* motor neuron G-protein signaling pathway were identified in our transcriptome analysis and shown in Table 2. All qPCR verification results were in agreement with our transcriptome RNA-seq results (Figure 4). In our qPCR verification results, the genes related to the promotion of the acetylcholine release pathway (*dop-1*, *egl-30*, and *unc-13*) and the gene related to the promotion of the neuropeptide release pathway (*kin-1*) showed the same trend that their expression was upregulated during PQ treatment but downregulated during PQ_AD treatment (treatments with PQ in present of adenosine) (Figures 4(a)–4(c) and 4(f)). Furthermore, the expression of these four genes treated with PQ was significantly higher than the CK and PQ_AD groups, respectively (*P* < 0.01). Interestingly, although the expression of the *ador-1* gene showed the same trend as *dop-1*, *egl-30*, *unc-13,* and *kin-1*, which is demonstrated in Figure 4(d), there were no significant differences between the three treatments including the CK, PQ, and PQ_AD groups (*P* > 0.05). This result suggested that the adenosine receptor was consistently and stably expressed regardless of whether the worms were PQ treated or not. In order to assess the dominance of the G-protein signaling pathway on adenosine protection effects, the gene (*goa-1*) that inhibited the above-mentioned pathways, including the acetylcholine release and neuropeptide release pathways, was also verified by qPCR. A completely opposite trend was observed for *goa-1* gene expression as was observed right after PQ treatment, and this gene decreased by 65.3% while increasing to 74.5 ± 8% after adenosine addition (Figure 4(e)).

### 3.6. Interaction Prediction Using the Adenosine Receptor Homolog Protein AdoR-1

**Figure 5:**
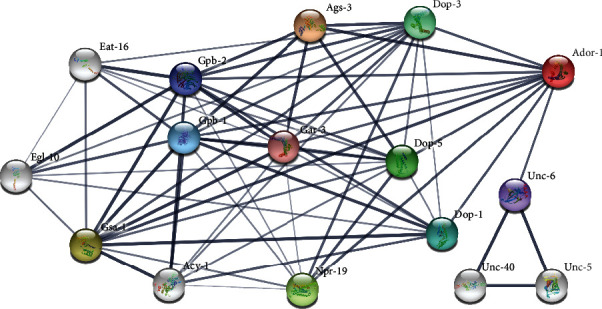
Protein-protein interaction network of AdoR-1. Line thickness indicates the strength of data support. The colored nodes associate with each other, and the majority of proteins interact with AdoR-1. Disconnected nodes are hidden from the network. The white nodes are second shell interactors. The confidence score in the current study was set to 0.900.

Using AdoR-1 as a seed, a total of 10 protein interaction maps were constructed using the STRING version 11.5 database. The interactions based only on text mining were also verified using the PubMed literature database. The results of this analysis of protein-protein interaction networks were highly connected network of molecules (Figure 5, PPI enrichment *P* value < 1.0 *e* − 16), and included 10 proteins. Detailed description of protein data is provided in Supplementary Table S3. The protein interaction network was visualized in the form of a graph network, with the protein molecules forming the nodes of the graph and the interactions forming the edges. Most of the proteins were at the center of the network with few molecules loosely arranged at the periphery. Some interactors were connected by multiple lines that indicate interactions derived from more than one source of information. Furthermore, 16 genes identified by qRT-PCR which were mentioned in Table 1 and Table 2 were also visualized for their protein interaction network with AdoR-1 (Supplementary Figure S1). The scores of *dop-1*, *dop-3*, and *dop-5* genes were higher than other genes, which were consistent with the interaction prediction of literature database.

### 3.7. Model of the Potential Mechanism of Adenosine Receptors in the Protection of Paraquat-Induced Oxidative Stress in *C. elegans*

**Figure 6:**
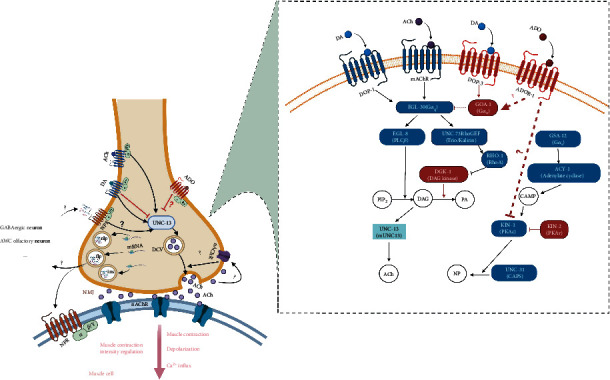
Model of potential mechanism of adenosine's protective effect against oxidative stress through AdoR-1. (a) schematic diagram of neuromuscular junction (NMJ) synaptic signaling molecular model. The path in black represents the promotion of the release of acetylcholine. The path in red represents the inhibition of the release of acetylcholine. The solid line represents a clear effect, while the dashed line represents possible direct or indirect effects. (b) a heterotrimeric G-Protein network that regulates the acetylcholine (ACh) signaling pathway in the neuromuscular junctions of *C. elegans* to control movement. G*α*q, G*α*12, and G*α*o control the production and destruction of the membrane-bound second messenger DAG. DAG stimulates the release of ACh and neuropeptides to control the contraction of integumental muscles. The fourth pathway is the G*α*s pathway, which converges with the other heterotrimeric G-protein pathways downstream of DAG to regulate the release of neuropeptides (NP). Note: mammalian homologs in parentheses are followed by *C. elegans* gene names. The graph is from a network of G-protein signaling pathways control neuronal activity in *C. elegans* and created using http://BioRender.com.

Based on our PPI network and qPCR data on members of the G-protein signaling pathway including *dop-1*, *egl-30*, *unc-13*, *kin-1,* and *goa-1*, the model demonstrated in Figure 6 was generated to demonstrate the potential mechanism of adenosine receptors in the protection from paraquat-induced oxidative stress in *C. elegans*. This model suggests that AdoR-1 may regulate dopamine via DOP-1 and molecules downstream of DOP-3 to modulate the content of DAG under paraquat-induced oxidative stress. Subsequently, AdoR-1 acts by reducing the content of DAG, thereby reducing the release of the neurotransmitter acetylcholine, and simultaneously modulating the release of neuropeptides by regulating the expression level of KIN-1 (PKAc).

### 3.8. Biochemical Assays between N2 and EG6890

**Figure 7:**
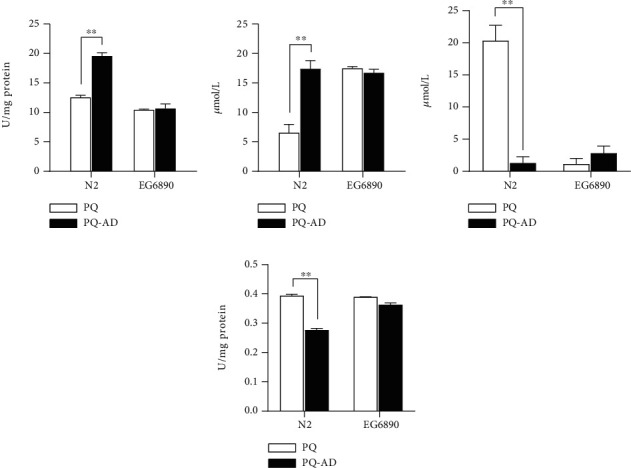
SOD, GSSG, GSH, and AChE activities of *C. elegans* N2 strain and EG6890 strain treated individually with PQ or PQ_AD. Three replicates were used per treatment. Differences compared to control group were considered significant at ^∗∗^*P* < 0.01.

The SOD, GSSG, GSH, and AChE activities of N2 wild-type nematodes have significant difference between PQ treatment and PQ_AD treatment. However, there were no significant differences between the PQ and PQ_AD in the EG6890 strain which *ador-1* gene was knockout (Figure 7). As for SOD activity of N2 strain, the PQ treatment group was significantly lower than that of PQ_AD treatment, indicating that adenosine might act on adenosine receptors to promote SOD activity then reduce oxidative stress for a significant protective effect. As for SOD activity of *ador-1* gene knockout strain EG6890, whatever PQ or PQ_AD conditions, it has no significant difference with PQ treatment of N2 nematodes but lower than PQ_AD treatment of N2 nematodes (Figure 7(a)). Similar results were found in GSSG and GSH biochemical assays (Figures 7(b) and 7(c)). The content of oxidized glutathione (GSSG) in the PQ treatment of N2 nematodes was significantly lower than that in the PQ_AD treatment. As for the content of reduced glutathione (GSH), that of PQ treatment of N2 nematodes was much higher than that of PQ_AD treatment and is also significantly higher than the two treatment groups of EG6890 strain. In Figure 7(d), AChE activity of N2 nematodes treated with PQ_AD was lower than that of PQ treatment, which indicated adenosine added could reduce the release of Ach after paraquat-treated. Obviously, whatever PQ or PQ_AD treatment, AChE activity of *ador-1* gene knockout strain EG6890, therefore, has no significant difference with PQ treatment of N2.

## 4. Discussion

Adenosine signaling based on the adenosine receptor induced by caffeine has been shown to partially extend the lifespan of *C. elegans* [21]. By tracing the progress of cell death and apoptosis in paraquat-poisoned *C. elegans*, adenosine was found to be protective of this oxidative stress [7]. In the present study, we carried out transcriptome analyses to explore the protective effect of adenosine exposure with paraquat in *C. elegans*.

Keeping an FDR ≤ 0.001 and an FC value ≥2 as the thresholds using our transcriptome data, CK vs. PQ revealed 2,269 differential genes, with 1,878 being upregulated and 391 being downregulated. In contrast to this, PQ vs. PQ_AD comparison yielded 1,188 differential genes, 213 of which were upregulated, and 975 were downregulated. As an important molecule, it seems that adenosine has a direct impact on kinds of genes that regulate their expression during paraquat exposure. Although numbers of differential expression genes decreased in presence of adenosine in paraquat-exposed *C. elegans*, 922 coregulated genes should be critical for exploring the relationship among the protective effect of adenosine and paraquat-induced oxidative stress (Figure 1). Furthermore, GO enrichment analysis comparing the CK vs. PQ vs. PQ_AD groups showed that the differentially expressed genes were mainly concentrated in gene family related to neural function (Figure 2). Based on previous studies [8, 22], these high-frequency gene families (listed in Supplementary Table S2), including dopamine neurotransmitter receptor genes (*DOP*), neuropeptide-related genes (*FLP*, *INS*, and *NLP*), neuropeptide receptor genes (*NPR*), and genes related to acetylcholine release (*UNC*), seemed to be relevant among those identified, based on their gene functions.

qRT-PCR verification of related key differential genes in the neurotransmitter signaling pathway was also shown to have a protective effect that may be related to the release of neuronal substances. Eleven genes in the neuropeptide gene family dominated by the *DOP*, *FLP*, and *NLP* gene families were significantly differentially expressed (Figure 3). These key genes have the functions of inducing defense responses and regulating neural Ca^2+^ levels [23].

Meanwhile, based on our transcriptome analysis and qPCR verification, *C. elegans* motor neuron G-protein signaling pathways involving *dop-1*, *egl-30*, *unc-13*, *kin-1*, and *goa-1* may play important roles in regulating the protective effect of adenosine (Table 2, Figure 4).

Interestingly, there were no significant expression changes for the *ador-1* gene between the PQ and PQ_AD groups, although a large number of genes were found to be downregulated after the addition of adenosine in the PQ group (Figure 4). This finding suggested that the adenosine receptor homolog AdoR-1 played a consistent role in the protective effect of adenosine regardless of whether worms were paraquat-treated or not.

It is well known that dopamine coregulates neuronal activity in *C. elegans* through two classes of GPCRs, including D1-like (DOP-1, DOP-5) and D2-like (DOP-3), as is demonstrated in Figure 5 and Supplementary Figure S1. In PPI network, it seems that the *dop-1*, *dop-3*, and *dop-5* genes have a higher score than other genes. DOP-1 and DOP-3 antagonize movement by acting on the same motor neurons, which coexpress receptors and do not have postsynaptic connections with dopaminergic neurons [24]. Moreover, DOP-1 may function through the G*α*q signaling pathway encoded by *EGL30* and *EAT-16* to regulate behavioral choice in cholinergic neurons, while DOP-3 may act through the G*α*o signaling pathway encoded by *goa-1* and *egl-30* to regulate behavioral choice in GABAergic neurons, RIC neurons, and SIA neurons [25]. Our quantitative results showed that the expression levels of *dop-1* and *dop-3* were both upregulated under paraquat-induced stress. In other words, *dop-1* was upregulated, 4.12 fold relative to control, and *dop-3* was upregulated, 1.15 fold relative to control. Clearly, the pathway of promoting the release of acetylcholine represented by *dop-1* was dominant under paraquat-induced stress. In addition, *GOA-1*, which is the downstream effector molecule of DOP-3, was expressed to only 0.35 fold of the CK group under paraquat-induced stress. Therefore, this indirectly indicated that the acetylcholine-promoting pathway represented by *dop-1* was dominant to *dop-3,* which represents the inhibition of the release of acetylcholine under paraquat-induced stress. Subsequently, the relative expression of *dop-1* and *dop-3* in the PQ_AD group was significantly reversed compared to the PQ group (*P* < 0.01). It seems that the activation of AdoR-1 reduced the content of DAG by regulating dopamine via DOP-1 and the molecular pair downstream of DOP-3, thereby reducing the release of the neurotransmitter acetylcholine. It is well known that the excessive release of acetylcholine is inherently toxic and will cause muscle cells to continue to be rigid [26]. This might explain why the morphology of *C. elegans* treated with paraquat have been observed to be rigid and transparent, but this phenotype yielded a longer protective effect with preconditioning with adenosine, as shown in our previous study [7].

Many of the proteins that regulate activity at the neuromuscular junction (NMJ) participate in a network of heterotrimeric G-protein signaling pathways controlling the release of synaptic vesicles and/or dense-core vesicles (DCVs) (Figure 6). There are at least four heterotrimeric G-proteins (G*α*q, G*α*12, G*α*o, and G*α*s) that act within motor neurons to control the activity of NMJs. The G*α*q, G*α*12, and G*α*o pathways converge to control the production and destruction of the lipid-bound second messenger diacylglycerol (DAG) at sites of neurotransmitter release. DAG acts via at least two effectors, mUNC13 and PKC, to control the release of both neurotransmitters and neuropeptides from motorneurons. The G*α*s pathway converges with the three other heterotrimeric G-protein pathways downstream of DAG to regulate neuropeptide release. Released neurotransmitters and neuropeptides then act to control the contraction of the body-wall muscles to control *C. elegans* locomotion [27].

Another mode of AdoR-1 action may be through regulating the expression level of KIN-1 (PKAc) and regulating the release of neuropeptides. Corresponding to the human homologous cAMP-PKAc pathway, the *kin-1* gene can activate cAMP-dependent protein kinase activity and is an ortholog of human PRKACA (protein kinase cAMP-activated catalytic subunit alpha). The increase of its expression level can promote an increase in neuropeptide levels. Similarly, the expression of *kin-1* in the PQ group was shown to be upregulated 3.23 fold compared to the CK group, while the average expression in the PQ_AD group was only 1.46 fold compared to the CK group. Adenosine treatment reduced this expression of *kin-1* to close to 50%. As is well known, the release of neuropeptides as a regulator can change the frequency of muscle oscillations in nematodes, thereby controlling the speed and direction of their movement. In contrast, this behavior is related to nociception and feeding. Therefore, another mode of AdoR-1 action may be through regulating the expression level of *kin-1* (PKAc) and regulating the release of neuropeptides, thereby alleviating the continuous contraction of muscle cells, reducing nociceptive perception and exercise frequency, thus achieving a protective effect, as is shown in Figure 6.

Based on the above, the protective mechanism of adenosine against oxidative stress in *C. elegans* may be through the continuous activation of the adenosine receptor homolog AdoR-1, the reduced release of acetylcholine, and the expression of key genes in the neuropeptide signaling pathway, acting to reduce acetylcholine. The release of neurotransmitters and neuropeptides relieves the toxicity of excess acetylcholine, and in body wall muscles, acetylcholine causes muscle contractions, slowing exercise frequency and nociceptive perception.

Here, we provide a model (Figure 6) of the potential mechanism of the protective function of AdoR-1 in which the core key molecules are the UNC-13 protein, DAG, and KIN-1. Although the mRNA transcript levels were verified to indicate that the genes related to the promotion of acetylcholine and neuropeptide release had a reduced expression after the addition of adenosine, more direct evidence supporting this mechanism is needed, including further exploration at the protein level and accurate measurement of the content of DAG at physiological and biochemical level. Furthermore, this study was only of a single homologous adenosine receptor in *C. elegans,* and studies on the four adenosine receptors (A1R, A2AR, A2BR, and A3R) in humans will benefit this study's findings on the evolution of paraquat-induced stress modulation.

Furthermore, in order to identify the potential factors involved in the protective mechanism of adenosine against oxidative stress, some biochemical assays were carried out between wild-type strain N2 and *ador-1* gene knockout strain EG6890. It is known that glutathione is an important antioxidant substance in cells; oxidized glutathione (GSSG) and reduced glutathione (GSH) are converted into each other under the catalysis of enzymes [28]. The content of glutathione was combined with the analysis of SOD activity, and it was speculated that under oxidative stress conditions. GSH reduces harmful oxidative substances under the catalysis of enzymes, reduces its toxicity, and is oxidized to GSSG itself. The effect of glutathione under oxidative stress conditions requires SOD enzyme to convert ROS into hydrogen peroxide first. As results were demonstrated in Figure 7, the SOD activity of N2 treated with PQ is relatively low, which may lead to reactive oxygen species (ROS) which could not be processed by SOD timely, and GSH in this group of nematodes could not play an antioxidant role. However, the ratio of GSH/GSSG in the PQ_AD treatment of N2 was low, probably because adenosine acted on adenosine receptors and produced much more SOD. Therefore, SOD could convert a large amount of ROS into hydrogen peroxide, which was further reduced by GSH and protect nematodes lastly.

The *ador-1* gene-knockout strain EG6890, the ratio of GSH/GSSG in the body is also low, indicating that GSH in the body is involved in the reduction reaction of ROS. However, combined with the results of Figure 7(a), the activity of SOD is not high, suggesting that adenosine receptor knockout may lead to the opening of other antioxidant pathways, and ROS is initially reduced through other pathways, and then further reduced by GSH.

Reduced glutathione (GSH) is considered to be one of the most important scavengers of ROS, and its ratio with oxidized glutathione (GSSG) may be used as a marker of oxidative stress. And the reduced release of acetylcholine appears during the adenosine added after paraquat treated in wild-type nematodes. Therefore, the SOD, GSH: GSSG ratio and AChE activity illustrated in Figure 7, were all matching and proving the adenosine's protective effect on oxidative stress through the adenosine receptor.

## 5. Conclusion

Taken together, our findings showed that genes from four classes involved in *DOP*, *FLP*, *NLP*, and *NHR* were differentially expressed when *C. elegans* worms were exposed to paraquat in the presence or absence of adenosine, and this study was the first to show the interaction between adenosine and neuronal G-protein signaling pathways in *C. elegans*. Furthermore, our research revealed that the protective effect of adenosine in terms of the oxidative stress induced by paraquat was mainly regulated by AdoR-1, and that several novel genes such as *dop-1*, *egl-30*, *unc-13*, *kin-1*, and *goa-1*, which were involved in key G-protein signaling pathways of the *C. elegans* adenosine receptor, deserve additional research. Additionally, some biochemical factors including SOD, GSSG, GSH, and AChE were identified for potential relationship with protective effect of AdoR-1 between wild-type strain N2 and *ador-1* gene-knockout strain EG6890. Our study provides new insights into the biological basis of the action of the adenosine receptor via RNA-seq and contributes to a better understanding of the mechanism of the adenosine receptor.

## Figures and Tables

**Table 1:** Summary of selected genes differentially expressed in CK vs. PQ and PQ vs. PQ_AD identified by GO enrichment. Gene families or functional groups with potential functions in *C. elegans* are emphasized. Gene ID in transcriptome data and WormBase ID are indicated for each gene. qRT-PCR primers and PCR parameters for differential genes in this study are also indicated.

Genes ID	Symbol	WormBase ID	Description	Primer sequence (5′-3′)	Size (bp)	Procedure
179347	*Dop-2*	WBGene00020506	DOPamine receptor	F:ACGGCGACTGGTATCTAGGA R:TTGGTAAAGAGACTGCACGGT	129	95°C for 60 seconds; 40 cycles of 95°C for 15 seconds; 40 cycles of 60 °C for 60 seconds
188499	*Dop-3*	WBGene00020506	DOPamine receptor	F:CCAGCGGATGGAATAAGCCAAGAC R:TGGCAACCAACAGAAGAGGAACAC	131	
179571	*Dop-5*	WBGene00064732	DOPamine receptor	F:ATCGCAGACACAGAAAGAGCCAAG R:CGGGGTTGCTGGTGGAGTATTTAC	103	
175462	*Nhr-6*	WBGene00003605	Nuclear hormone receptor family	F:CGCTGTGTGCAATGATCGAG R:CTTGTCGATTGGGCAGGTCT	139	
178024	*Nhr-67*	WBGene00003657	Nuclear hormone receptor family	F:GCCACCACAACAACCCACTCC R:GCTACTCGTCGCATCATCCTCTTC	133	
181221	*Flp-3*	WBGene000001446	FMRF-like peptide	F:CCCAATCACCTCATCCTGCTCTTC R:CGCATTGTTCCCAGTGGAGACC	92	
178232	*Flp-9*	WBGene00001452	FMRF-like peptide	F:CTTGTCGCCTGTATTGCAGC R:TCCTTTCCACAGAACTCGGC	80	
180587	*Flp-18*	WBGene000001461	FMRF-like peptide	F:GTTCGACAAGCGCAGTGAAG R:CCCTGCGTAATGCTCTTCCA	109	
185978	*Nlp-3*	WBGene000003741	Neuropeptide-like protein	F:ATCGTCGCTTGCTTGGTCTTACTC R:ACGGGTTGACAGCTCGTTTTCC	127	
177387	*Nlp-16*	WBGene00003754	Neuropeptide-like protein	F:CGTGCCGGATCCAGTCACAAAG R:GAGTCGTTCTGCGTGGTTTCTCC	83	
176570	*Nlp-21*	WBGene00003759	Neuropeptide-like protein	F:CTTGTCATGGTGCTCAACGC R:ACCTCGCTTGTAGTCGTCAC	138	
179535	*Act-1*	WBGene00000063	Actin	F:CTTCCCTCTCCACCTTCCAAC R:GCTGGTGGTGACGATGGTTT	144	

**Table 2:** Summary of G-protein signaling genes differentially expressed in CK vs. PQ and PQ vs. PQ_AD. Genes are organized into categories based on molecular function (KOG or other description, http://www.wormbase.org). Gene families or functional groups with potential functions in motor neurons in *C. elegans* are emphasized. Gene IDs in transcriptome data are indicated for each gene. qRT-PCR primers for genes related to G-protein signaling pathways and PCR parameters in this study are also indicated.

Genes ID	Symbol	WormBase ID	KOG (other description)	Primer sequence(5′-3′)	Size (bp)	Procedure
180714	*Dop-1*	WBGene00001052	7 transmembrane receptor, enables dopamine neurotransmitter receptor activity, coupled via Gs	F:CCTTGTTTCTTTGGCCGTGT R:GAACGGCCAGTATCCCAAGA	91	95°C for 60 seconds; 40 cycles of 95°C for 5 seconds; 40 cycles of 60°C for 30 seconds
171751	*Egl-30*	WBGene00001196	G protein subunit Galphaq/Galphay, small G protein superfamily	F:ACTCGCATCTCGCTGACTAC R:CGCGCACGTAAAATGAGAGT	140	
172497	*Unc-13*	WBGene00006752	Neurotransmitter release regulator, UNC-13	F:TGTGACCACTTTGGAACCCC R:TAGAACTGCATCGGCTTCCG	121	
174890	*Ador-1*	WBGene00011878	7 transmembrane receptor	F:GCGCTCACGGATTTTCTAGC R:CACAAAGAGGCACCCGTAGA	99	
172505	*Goa-1*	WBGene00001648	G-protein alpha subunit (small G protein superfamily)	F:TTTTGCGAGCCATGAGCAAC R:TCTGTGTCCTCCATTCGTGC	103	
3565407	*Kin-1*	WBGene00002189	cAMP-dependent protein kinase types I and II, regulatory subunit	F:AAGCATAAGCAGTCGGGCAA R:GATAGCCTGGAGAATGCGCT	114	

## Data Availability

The sequencing data used to support the findings of this study have been deposited in the BioProject database as PRJNA809866, and the website was http://www.ncbi.nlm.nih.gov/bioproject/809866.

## Supplementary Materials

Supplementary Materials Figure S1: protein–protein interaction network of the genes identified by qRT-PCR in this study. Table S1: summary of data quality assessment. Table S2: high-frequency gene families and their functions in the five GO categories. Table S3: prediction of protein ADOR-1-associated protein.
