# Supplementary material for: AdoR-1 (Adenosine Receptor) Contributes to Protection against Paraquat-Induced Oxidative Stress in Caenorhabditis elegans
Source: Oxid Med Cell Longev. 2022 Dec 22;2022:1759009. doi: 10.1155/2022/1759009 (PMC9800083; doi:10.1155/2022/1759009)

**Fig. S1** **Protein–protein interaction network of the genes identified by qRT-PCR in this study**. Involved genes in this figure were all selected from Table 1 and Table 2. Line thickness indicates the strength of data support. The colored nodes associate with each other and the majority of proteins interact with AdoR-1. Disconnected nodes are also presented. The confidence score in the current study was set to medium (0.400).


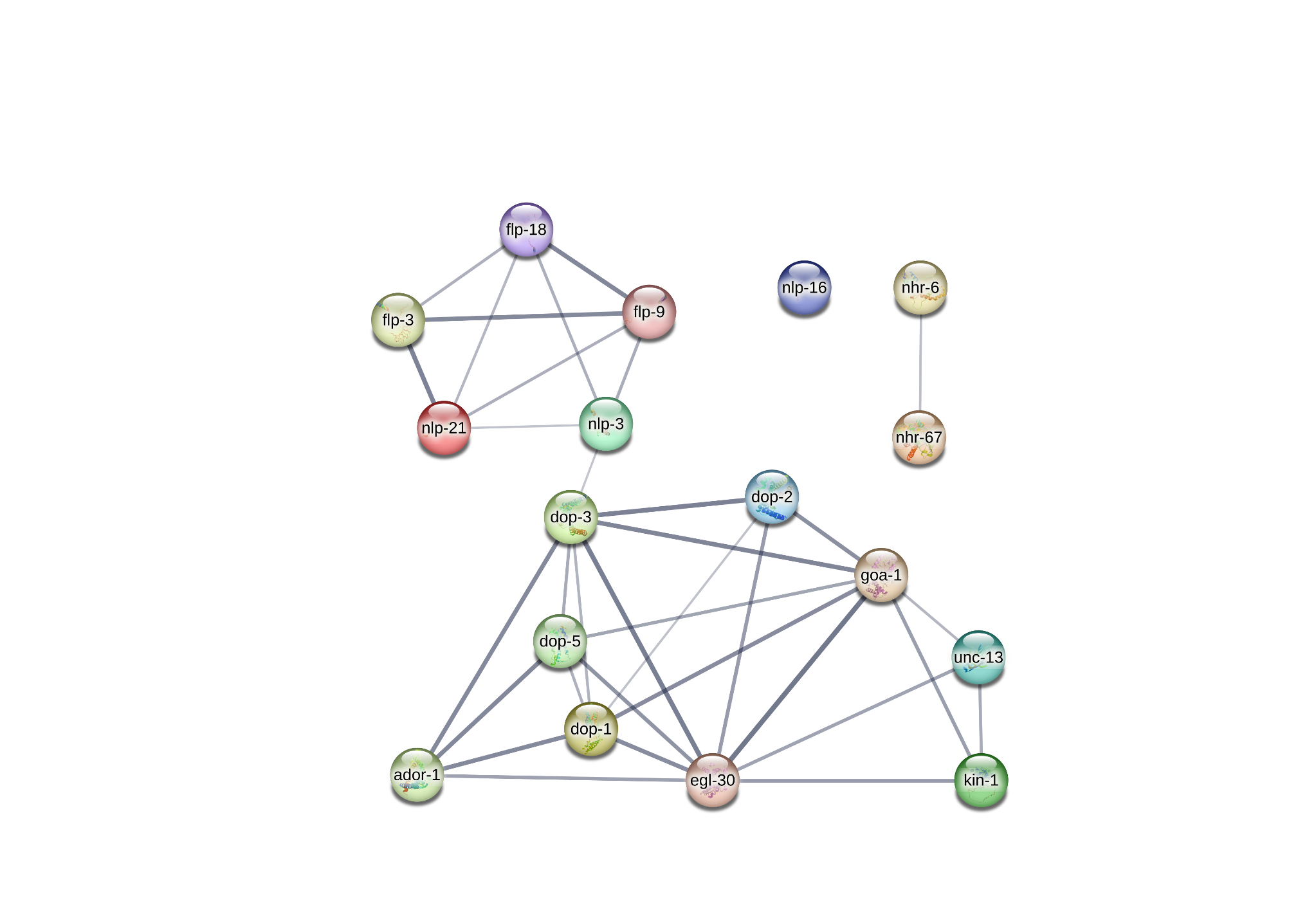

Supplement: Supplementary Materials — Figure S1: protein–protein interaction network of the genes identified by qRT-PCR in this study. Table S1: summary of data quality assessment. Table S2: high-frequency gene families and their functions in the five GO categories. Table S3: prediction of protein ADOR-1-associated protein. [file 1759009.f1.zip › Supplementary Figure S1 (1).docx]
