# Supplementary material for: AdoR-1 (Adenosine Receptor) Contributes to Protection against Paraquat-Induced Oxidative Stress in Caenorhabditis elegans
Source: Oxid Med Cell Longev. 2022 Dec 22;2022:1759009. doi: 10.1155/2022/1759009 (PMC9800083; doi:10.1155/2022/1759009)
Supplement: Supplementary Materials — Figure S1: protein–protein interaction network of the genes identified by qRT-PCR in this study. Table S1: summary of data quality assessment. Table S2: high-frequency gene families and their functions in the five GO categories. Table S3: prediction of protein ADOR-1-associated protein. [file 1759009.f1.zip › Supplementary Table S1.docx]

Table S1 Summary of data quality assessment

| Items | CK | PQ | PQ_AD |
| --- | --- | --- | --- |
| Total Raw Reads (M) | 50.65 | 46.98 | 54.01 |
| Total Clean Reads (M) | 44.05 | 40.41 | 46.39 |
| Total Clean Bases(Gb) | 6.61 | 6.06 | 6.96 |
| Clean Reads Q20(%) | 95.62 | 95.76 | 95.84 |
| Clean Reads Q30(%) | 85.65 | 86.06 | 86.28 |
| Clean Reads Ratio(%) | 86.98 | 86.02 | 85.89 |
