# Supplementary material for: AdoR-1 (Adenosine Receptor) Contributes to Protection against Paraquat-Induced Oxidative Stress in Caenorhabditis elegans
Source: Oxid Med Cell Longev. 2022 Dec 22;2022:1759009. doi: 10.1155/2022/1759009 (PMC9800083; doi:10.1155/2022/1759009)
Supplement: Supplementary Materials — Figure S1: protein–protein interaction network of the genes identified by qRT-PCR in this study. Table S1: summary of data quality assessment. Table S2: high-frequency gene families and their functions in the five GO categories. Table S3: prediction of protein ADOR-1-associated protein. [file 1759009.f1.zip › Supplementary Table S2.docx]

Table S2 High-frequency Gene families and their functions in the five GO categories

| Gene Family | Contained Genes | Gene Function |
| --- | --- | --- |
| abu | *abu-1/4/6/7/8/9/10/11/15* | Involved in endoplasmic reticulum unfolded protein response. |
| daf | *daf-5/7/12/28/37* | Regulation of gene expression, negative regulation of entry into the dauer phase, development and social behavior of dauer larvae, protein entry into the nucleus, regulation of synaptic growth at neuromuscular junctions, response to bacteria |
| dop | *dop-2/3/5/6* | Food-responsive dopamine receptor signaling pathway, adenylate cyclase inhibitory dopamine receptor signaling pathway |
| flp | *flp-1/3/5/6/8/9/11/13/14/17/18/19* | Neuropeptide signaling pathway, sleep, movement, mating regulation, action potential regulation, spawning regulation |
| gcy | *gcy-9/11/15/19/21/22* | Neuronal action potential regulation, cellular pH response, detection of CO2 |
| glr | *glr-1/4/5* | Movement, learning, memory, regulation of postsynaptic membrane potential, transmitter-gated ion channels |
| ins | *ins-9/19/24/26/28/30* | Enable hormone activity. signal transduction |
| nas | *nas-4/6/8/13/14/15* | Involved in pharyngeal pump development |
| nhr | *nhr-6/49/58/67/68* | Involved in determining adult lifespan, positive regulation of RNA polymerase II promoter transcription, stress regulation of fatty acid metabolism, gonadal morphogenesis, positive regulation of cell cycle arrest |
| nlp | *nlp-3/6/9/11/13/15/16/18/21/35/37/42/48/50/51* | Involved in neuropeptide signaling pathway |
| npr | *npr-16/18/25/29/34* | Enable G protein-coupled receptor activity and peptide binding activity, involved in G protein-coupled receptor signaling pathway |
| pqn | *pqn-2/54/57/74/90* | GABAergic synaptic transmission, cholinergic synaptic transmission, striated muscle myosin thick filament assembly |
| ttr | *ttr-1/14/21/22/26/27/28/29* | Involved in the defense response to Gram-negative bacteria, affected by daf-2, daf-16 and other genes |
| unc | *unc-13/17/25/53/54/73/82* | Enable calmodulin binding activity and syntaxin-1 binding activity, involved in several processes, including regulation of pharyngeal pumping; regulation of reproductive process; and synaptic vesicle exocytosis |
