# Supplementary material for: AdoR-1 (Adenosine Receptor) Contributes to Protection against Paraquat-Induced Oxidative Stress in Caenorhabditis elegans
Source: Oxid Med Cell Longev. 2022 Dec 22;2022:1759009. doi: 10.1155/2022/1759009 (PMC9800083; doi:10.1155/2022/1759009)
Supplement: Supplementary Materials — Figure S1: protein–protein interaction network of the genes identified by qRT-PCR in this study. Table S1: summary of data quality assessment. Table S2: high-frequency gene families and their functions in the five GO categories. Table S3: prediction of protein ADOR-1-associated protein. [file 1759009.f1.zip › Supplementary Table S3.docx]

**Table S3 Prediction of protein ADOR-1-associated protein**

| **Gene** | **Predicted Functional** | **Score** |
| --- | --- | --- |
| *ags-3* | Activator of g protein signalling; Protein AGS-3, isoform a | 0.944 |
| *gsa-1* | G protein, Subunit Alpha; Protein GSA-1 (*gsa-1*) mRNA, complete cds | 0.912 |
| *npr-19* | NeuroPeptide Receptor family; Protein NPR-19 (*npr-19*) mRNA, complete cds | 0.844 |
| *dop-5* | DOPamine receptor; Protein DOP-5 (*dop-5*) mRNA, complete cds; Belongs to the G-protein coupled receptor 1 family | 0.817 |
| *dop-3* | Dopamine receptor 3; Receptor for dopamine. The activity of this receptor is mediated by G proteins which activate adenylyl cyclase. In terms of antagonist responses, would be classed with the D2-like dopamine receptor group. Mediates the effect of dopamine on the inhibition of locomotion. Acts as an antagonist of *dop-1* | 0.811 |
| *dop-1* | DOPamine receptor; Protein DOP-1, isoform a; Belongs to the G-protein coupled receptor 1 family | 0.809 |
| *gpb-1* | Guanine nucleotide-binding protein subunit beta-1; Guanine nucleotide-binding proteins (G proteins) are involved as a modulator or transducer in various transmembrane signaling systems. The beta and gamma chains are required for the GTPase activity, for replacement of GDP by GTP, and for G protein- effector interaction. In the early embryo, controls the magnitude of the forces acting on centrosomes but is not required for generating asymmetric forces | 0.8 |
| *gpb-2* | Guanine nucleotide-binding protein subunit beta-2; Guanine nucleotide-binding proteins (G proteins) are involved as a modulator or transducer in various transmembrane signaling systems. The beta and gamma chains are required for the GTPase activity, for replacement of GDP by GTP, and for G protein- effector interaction | 0.774 |
| *unc-6* | Netrin unc-6; Component of an extracellular matrix cue that guides dorsoventral migrations on the epidermis. Required for the guidance of pioneer axons and migrating cells along the body wall. During gonad morphogenesis, involved in distal tip cell (DTC) migration from the dorsal side of the hermaphrodite body to the midbody to allow for formation of gonad arms. Its association with either *unc-40* or *unc-5* receptors will lead to axon attraction or repulsion, respectively. Involved in the positioning of ray 1, the most anterior ray sensilium, in the male tail | 0.767 |
| *gar-3* | Muscarinic acetylcholine receptor *gar-3*; The muscarinic acetylcholine receptor mediates various cellular responses, including inhibition of adenylate cyclase, breakdown of phosphoinositides and modulation of potassium channels through the action of G proteins. Primary transducing effect is Pi turnover (By similarity). Enhances the release of the neurotransmitter acetlycholine in cholinergic motor neurons, which in turn positively feeds back to depolarize body wall muscles and allows for the maintenance of normal body posture and locomotion | 0.755 |
